# Modeling Zinc Absorption in the Adult Population of Colombia: Insights for Nutritional Evaluation and Intervention Strategies

**DOI:** 10.1007/s12011-024-04180-x

**Published:** 2024-05-13

**Authors:** José Alexander Alvarez-Bustamante, Angélica María Muñoz

**Affiliations:** Faculty of Nutrition and Food Sciences, CES University, Medellín, Colombia

**Keywords:** Dietary reference intake, Mathematical model, Micronutrients, Phytate, Survey, Zinc absorption

## Abstract

Zinc is a vital trace element, yet its deficiency is common in various populations. This study addresses the gap in understanding zinc intake and its relationship with key nutritional parameters in a Colombian population. We analyzed data from 12,987 individuals, focusing on the daily intake of zinc, phytate, protein, and calcium, and used the phytate/zinc molar ratio as an input parameter in the Miller et al. (2013) model. This model was employed to estimate the total absorbed zinc (TAZ) and the fractional absorption of zinc (FAZ). Our findings highlight a general trend towards insufficient intake compared to the standards of the Institute of Medicine (IOM) and Colombia, with a significant percentage of the population falling below the estimated average requirement (EAR) and recommended daily allowance (RDA) for zinc, underscoring the need for targeted nutritional strategies. Our study contributes to a broader understanding of zinc nutrition and public health implications in Colombia, providing a basis for future dietary guidelines and health interventions.

## Introduction

The essentiality of zinc for optimal functioning is underscored by its role in over 2800 human proteins, encompassing catalytic, structural, or regulatory activities [1, 2]. Inadequate zinc intake, affecting human health, can manifest in impaired immune function, delayed growth and development, skin problems, loss of appetite and weight, and impaired reproductive function [3]. Primary dietary sources of zinc include meat and shellfish [4]. Additionally, dairy products and whole grains are significant sources [5]. Notably, despite fortification efforts, cereals generally exhibit lower zinc levels [6]. Plant-based diets, compared to animal-derived meals, are associated with lower zinc bioavailability [7]. Oral zinc ingestion and fractional absorption range from 16 to 50%, with absorption decreasing with increasing intake and vice versa [8].

Evidence suggests that low- and middle-income nations face public health risks due to zinc deficit [9]. Regions like Latin America and the Caribbean, marked by limited biochemical investigations, inadequate zinc intake, and high rates of stunted development, show a notable prevalence of zinc insufficiency among children under the age of 6 [10]. Zinc deficiency, once considered prevalent mainly in low- and middle-income countries, now extends to developed nations [11, 12]. Additionally, populations such as those with inflammatory bowel disease, older adults, vegans, vegetarians, and individuals with disorders like celiac disease or acrodermatitis enteropathica are susceptible to illnesses resulting from zinc deficiency [13, 14].

Plasma or serum contains less than 1% of the body’s total zinc content, typically falling between 80 and 120 µg/dL (approximately 12 to 18 µmol/L) [13, 15]. Prior studies conducted in Colombia, focusing on children between the ages of 1 and 4, revealed blood zinc levels [16, 17]. Factors influencing plasma zinc levels include dietary habits, socio-demographic characteristics, and metabolic markers such as glucose homeostasis [18]. This variability in plasma zinc levels complicates the reliable assessment of zinc status through serum or plasma tests [18]. In Colombia, one of the highest incidences of zinc insufficiency affects 43.3% of children under the age of 5 [9].

Children, with heightened physiological needs during growth, are particularly vulnerable to zinc deficiency [13, 19]. Assessing zinc status in older age groups is crucial for evaluating overall community nutritional health. Dietary data from the National Nutritional Situation Survey 2005 (ENSIN 2005) in Colombia provided insights into zinc intake relative to the recommended amounts [16]. Global dietary guidelines for zinc vary due to methodological differences, endpoints, evidence types, expert opinions, and process assumptions [20, 21]. Recommended dietary allowances (RDA) for zinc in the Colombian population are 14 mg/day and 8 mg/day for healthy men and women, respectively. Mean zinc intake in healthy individuals varies by age, sex, and life stages such as pregnancy and lactation. Phytate, oxalic acid, polyphenols, and various minerals, including calcium, iron, copper, and cadmium, as well as proteins, play pivotal roles in nutrient interactions [13, 22]. Even small quantities of animal protein may improve zinc absorption from legumes, as zinc from protein sources demonstrates effective absorption [22]. Plants store phosphorus predominantly as phytate, a compound with a strong affinity for binding zinc and other minerals. Consequently, the active transporters within the lumen of the small intestine are rendered incapable of facilitating zinc transfer. Grain, legumes, nuts, seeds, and whole-grain cereals are all potential sources of phytate [19]. In order to align dietary practices with current nutritional insights [23], a critical evaluation of the influence of phytate on zinc absorption becomes important, particularly if the offered or suggested meals include it [13]. This assessment is crucial for optimizing nutritional strategies and fostering a comprehensive understanding of the intricate dynamics involved in mineral absorption. The most effective approach for examining zinc absorption in the human body has been using a zinc isotope marker in vivo [24, 25], but this method is expensive and requires ethical concerns when involving human subjects [26]. Therefore, different strategies have been undertaken to enhance our understanding of zinc transport and absorption, including in vitro experiments [27] and mathematical models [13, 28–31].

Miller and colleagues developed a mathematical model capable of computing the quantity of absorbed zinc while accounting for dietary factors such as calcium, iron, protein, and phytate [29–31]. This model was constructed based on a statistical analysis of secondary data and biochemical research. To effectively compare reported recommended intakes with those advised by the Institute of Medicine (IOM), Armah [23] analyzed data from NHANES 2009–2010. Miller et al. [31] proposed a mathematical model that offers an innovative and cost-effective approach to determine dietary zinc bioavailability. This model represents a significant tool for assessing zinc absorption from various diets, underscoring its utility in nutritional research. Given its potential to assess the bioavailability of zinc in the foods consumed by individuals, this model may serve as a valuable supplementary tool for studies based on nutritional surveys in communities [31].

Our study aimed to utilize dietary information from Colombia’s National Nutritional Situation Survey 2005 (ENSIN 2005) to predict the extent of zinc absorption using the model developed by Miller et al. [31]. In comparison to biochemical investigations of zinc absorption, this technique offers a practical means to evaluate the applicability and impact of the established model. The utilization of mathematical models for zinc absorption in diverse populations can aid in comprehending their range and limits, identifying potential improvements, and gaining deeper insights into the physiology and metabolism of zinc in the human body.

## Materials and Methods

### Assessment of Daily Zinc Intake

In our study, we gathered information on the daily intake of zinc, calcium, and protein for each participant. Phytate levels were estimated using data from the ENSIN 2005 survey, supplemented by the FAO/IZiNCG 2018 PhyFoodComp tables [32].

We applied the model developed by Miller et al. [31] to calculate the fraction of absorbed zinc (FAZ) and total absorbed zinc (TAZ). This model includes critical variables such as total daily phytate (TDP), total daily zinc (TDZ), total daily calcium (TDC), and total daily protein (TDPr). The model effectively quantifies zinc absorption, aligning with the methodologies outlined in Armah [23].

We use the estimated average requirement (EAR) and recommended dietary allowance (RDA) as benchmarks for zinc intake, following the guidelines established by the IOM [20] and Colombian legislation [33]. The EAR indicates the daily intake required to meet the needs of half the population, while the RDA is set at 1.2 times the EAR [20]. We calculated EAR and RDA for Colombia (EAR_M and RDA_M) based on the ENSIN 2005 survey data and the FAZ from the Miller et al. [31] model, using Eqs. 1 and 2. In these equations, the required absorbed zinc (RAZ), according to the IOM, is applied, which is 3.84 mg/day for men aged 19 and over and 3.30 mg/day for women aged 19 and over [20].

1 $$EAR\_M\left(\frac{\mathrm{m}\mathrm{g}}{\mathrm{d}\mathrm{a}\mathrm{y}}\right)=\frac{RAZ}{FAZ\left(\%\right)}\times 100\%$$

2 $$RDA\_M\left(\frac{\mathrm{m}\mathrm{g}}{\mathrm{d}\mathrm{a}\mathrm{y}}\right)=1.2\times EAR\_M\left(\frac{\mathrm{m}\mathrm{g}}{\mathrm{d}\mathrm{a}\mathrm{y}}\right)$$

### Statistical Analysis

Statistical analyses were conducted using R software. The Shapiro-Wilk test was initially applied to assess data normality within each age and sex group, ensuring non-parametric tests were selected for non-normally distributed data. We summarized the data by reporting median values along with their corresponding 25th and 75th percentiles (Q1, Q3). Our emphasis was on assessing the adequacy of zinc intake across different age and sex demographics relative to established nutritional benchmarks.

The Wilcoxon one-sample test was utilized: to compare EAR_M and RDA_M against established EAR and RDA values; to compare TAZ with RAZ benchmarks; and to compare FAZ against the IOM’s zinc absorption rates. This test was selected for its capacity to assess whether the median of a sample significantly deviates from known benchmarks, providing a robust method for evaluating alignment with nutritional standards as well as zinc absorption metrics.

A significance threshold of *p* < 0.05 was adopted to indicate statistical significance across all tests, with a stricter threshold of *p* < 0.001 noted for particularly robust findings.

## Results

### Nutrient Intake by Age and Sex

Table 1 presents a summary of the median nutrient intake values, stratified by age and sex categories, which offers an insight into the dietary patterns of the study population. Our findings reflect the distribution of zinc, phytate, protein, and calcium intake within these demographic segments, as well as their respective phytate-to-zinc (P/Z) molar ratios, a marker for zinc bioavailability. We detailed the nutrient intake levels in the context of current nutritional benchmarks, with the aim of discerning the sufficiency of zinc intake among different demographic groups.

**Table 1:** Dietary zinc intake and related nutritional parameters

		Median (Q1, Q3)				
Age group (by years)	*n*	Zinc, mg/day	Phytate, mg/day	Protein, g/day	Calcium, mg/day	P/Z molar ratio
Men						
19–30	2754	8.1 (5.5, 11.4)	590.6 (320.4, 1040.1)	59.9 (42.4, 80.5)	317.3 (176.7, 555.6)	7.5 (4.2, 12.9)
31–50	2431	7.5 (5.1, 10.5)	570.9 (298.9, 976.4)	54.7 (40.0, 75.5)	304.1 (159.3, 513.9)	7.8 (4.2, 13.1)
51–70	867	6.6 (4.6, 9.6)	535.1 (287.7, 945.3)	48.4 (34.3, 65.9)	278.5 (134.8, 488.9)	8.4 (4.4, 15.4)
Total men	6052	7.6 (5.2, 10.7)	573.3 (306.8, 1002.0)	56.1 (40.0, 77.0)	308.0 (163.6, 530.0)	7.7 (4.2, 13.3)
Women						
19–30	3057	6.2 (4.2, 8.9)	458.5 (250.9, 773.2)	45.8 (32.4, 62.1)	285.0 (144.0, 500.6)	7.5 (4.1, 13.0)
31–50	2795	5.5 (3.7, 7.7)	409.5 (214.6, 713.5)	40.2 (28.7, 54.0)	255.1 (125.3, 461.3)	7.7 (4.2, 13.4)
51–70	1083	4.7 (3.1, 6.8)	410.2 (217.1, 701.8)	35.2 (23.8, 47.2)	230.6 (113.1, 436.2)	8.8 (4.6, 15.0)
Total women	6935	5.7 (3.8, 8.1)	430.2 (229.2, 741.0)	41.5 (29.4, 56.6)	266.4 (130.8, 475.7)	7.8 (4.2, 13.4)
Total population	12987	6.5 (4.3, 9.3)	487.0 (260.1, 845.6)	47.7 (33.2, 66.3)	284.7 (145.2, 499.3)	7.7 (4.2, 13.4)

Q1 and Q3 represent the 25th percentile (25%ile) and the 75th percentile (75%ile) values, respectively

### Zinc Intake Deficiency Across Demographics

More than half of the Colombian population, irrespective of age, consumes zinc at levels below the EAR and the RDA as per IOM standards (Table 2). Specifically, 62.2% of men aged 19–30, 66.9% of those 31–50, and 73.8% of those 51–70 have zinc intakes below the IOM EAR. This trend is more pronounced when considering the RDA, with 73.3% of men aged 19–30, 77.8% of those 31–50, and 84.5% of those 51–70 not meeting the standard. A sex-based analysis reveals that women have a slightly lower percentage of zinc intake inadequacy compared to men for the IOM EAR, with figures being 55.7%, 66.3%, and 75.0% for the respective age groups.

**Table 2:** Comparison of population percentage below EAR and RDA values according to IOM and Colombian standards, by age group

Age group (by years)	% below IOM EAR	% below IOM RDA	% below Colombia EAR	% below Colombia RDA
Men				
19–30	62.2	73.3	78.5	86.4
31–50	66.9	77.8	81.4	88.9
51–70	73.8	84.5	87.8	93.3
Total men	65.8	76.7	81.0	88.4
Women				
19–30	55.7	67.6	52.8	67.6
31–50	66.3	77.7	63.0	77.7
51–70	75.0	84.1	71.7	84.1
Total women	63.0	74.2	59.9	74.2

The percentages reflect the proportion of the population with zinc intake below the estimated average requirement (EAR) and recommended dietary allowance (RDA) according to the Institute of Medicine (IOM) [20] and Colombian nutritional standards [33]. No statistical tests were applied as the table presents descriptive data

When assessed against Colombian standards, the data show an even greater percentage of the population with inadequate zinc intake. The percentages of men aged 19–30, 31–50, and 51–70 below the Colombian EAR are 78.5%, 81.4%, and 87.8%, respectively, with similar trends observed in women (Table 2).

### Evaluation of Zinc Absorption Metrics

In evaluating the zinc absorption metrics detailed in Table 3, our analysis revealed pivotal insights into the nutritional zinc status of different demographics in the Colombian population.

**Table 3:** Comparison of total absorbed zinc (TAZ) with required absorbed zinc, and the fraction of absorbed zinc (FAZ) with zinc absorption according to IOM

Age group (by years)	Required absorbed zinc (RAZ) according to IOM, mg/day	TAZ, mg/day, median (Q1, Q3)	Zinc absorption according to IOM, %	FAZ, %		
				Median (Q1, Q3)	Mean ± SD	Min, max
Men						
19–30	3.84	2.5 (1.9, 3.2)	41	31.0 (25.0, 38.2)	32.0 ± 9.6	9.8, 69.1
31–50	3.84	2.4 (1.7, 3.1)	41	31.6 (25.6, 39.3)	32.8 ± 10.1	11.2, 84.8
51–70	3.84	2.1 (1.5, 2.9)	41	32.9 (25.9, 39.8)	33.4 ± 9.9	11.8, 73.4
Total men	3.84	2.4 (1.7, 3.1)	41	31.4 (25.4, 38.8)	32.5 ± 9.9	9.8, 84.8
Women						
19–30	3.30	2.2 (1.5, 2.9)	48	34.6 (28.8, 41.6)	35.4 ± 9.3	9.7, 71.3
31–50	3.30	2.0 (1.4, 2.6)	48	36.4 (29.8, 43.4)	36.8 ± 9.5	8.9, 71.0
51–70	3.30	1.7 (1.1, 2.4)	48	37.0 (30.5, 43.7)	37.4 ± 9.5	14.6, 63.5
Total women	3.30	2.0 (1.4, 2.7)	48	35.7 (29.5, 42.7)	36.2 ± 9.4	8.9, 71.3

Q1 and Q3 are the 25th and 75th percentile values, respectively. TAZ values were compared to the IOM’s RAZ using the Wilcoxon one-sample test, revealing a deficiency across all groups (*p* < 0.001). FAZ medians also fell below the IOM’s absorption rates for both men (41%) and women (48%), as shown by Wilcoxon one-sample test results (*p* < 0.001). SD, min, and max represent standard deviation, minimum, and maximum values for FAZ, respectively

The TAZ, expressed as a percentage of the median intake, was consistently lower than the RAZ according to IOM across all age groups and sexes (Table 3). For example, men aged 19–30 had a median TAZ of 2.5 mg/day, which is approximately 65.1% of the IOM’s required 3.84 mg/day. This shortfall was observed across all groups, indicating a widespread deficiency when compared to the IOM standards (*p* < 0.001, Wilcoxon one-sample test).

Regarding the FAZ, detailed in Table 3, we observed that the median percentage of zinc absorption was below the absorption rates recommended by the IOM, 41% for men and 48% for women (*p* < 0.001, Wilcoxon one-sample test). The distribution of FAZ values across the population shows a notable breadth, ranging from a minimum of 8.9% to a maximum of 84.8%. Although the mean FAZ was close to the median, suggesting aggregation towards central values, the Shapiro-Wilk test (*p* < 0.001) confirmed the data’s non-normal distribution, indicative of significant variability within the population.

### Zinc Intake Recommendation Analysis

The zinc intake recommendations derived from the model of Miller et al. [31] provide nuanced insights compared to the IOM and Colombian standards (Table 4). Notably, the median EAR_M and RDA_M values calculated from the Miller et al. model differ significantly from both IOM and Colombian recommended intakes for most age and sex groups, underscoring the potential for refined dietary guidelines based on these model outputs.

**Table 4:** Zinc intake recommendations of the IOM, Colombia, and from study data using model of Miller et al.

Age group (by years)	IOM, mg/day		Colombia, mg/day		Model of Miller et al., median (Q1, Q3), mg/day	
	EAR IOM	RDA IOM	EAR Col.	RDA Col.	EAR_M	RDA_M
Men						
19–30	9.4	11	12	14	12.4 (10.1, 15.3)	14.9 (12.1, 18.4)
31–50	9.4	11	12	14	12.2 (9.8, 15.0)	14.6 (11.7, 18.0)
51–70	9.4	11	12	14	11.7 (9.6, 14.8)^a^	14.0 (11.6, 17.8)^b^
Total men	9.4	11	12	14	12.2 (9.9, 15.1)	14.7 (11.9, 18.2)
Women						
19–30	6.8	8	6.5	8	9.5 (7.9, 11.5)	11.4 (9.5, 13.7)
31–50	6.8	8	6.5	8	9.1 (7.6, 11.1)	10.9 (9.1, 13.3)
51–70	6.8	8	6.5	8	8.9 (7.5, 10.8)	10.7 (9.1, 13.0)
Total women	6.8	8	6.5	8	9.3 (7.7, 11.2)	11.1 (9.3, 13.4)

Q1 and Q3 represent the 25th percentile (25%ile) and the 75th percentile (75%ile) values, respectively. The Wilcoxon one-sample test indicated statistical significance with *p* < 0.001 compared with IOM and Colombian recommendations, with exceptions noted

^a^For men aged 51–70 years, no significant difference was found in the comparison with EAR Col (*p* = 0.1581, Wilcoxon one-sample test)

^b^Although the median RDA_M for men aged 51–70 was 14.0, the same as the RDA Col, the Wilcoxon one-sample test revealed significant differences in the distribution of the data compared to the RDA Col (*p* < 0.001)

For men, EAR_M values exceeded the Colombian EAR across all age groups, showing statistical significance (*p* < 0.001, Wilcoxon one-sample test) except for men aged 51–70 years, where the difference was not statistically significant (*p* = 0.1581). This indicates a closer alignment between EAR_M and Colombian EAR for this age group, suggesting that the current EAR may be appropriate for these individuals. However, despite the median RDA_M being the same as the Colombian RDA for the 51–70 age group, significant statistical variation was detected (*p* < 0.001, Wilcoxon one-sample test).

In women, the scenario was more pronounced, with EAR_M and RDA_M significantly exceeding both IOM and Colombian recommendations across all age groups (*p* < 0.001, Wilcoxon one-sample test), pointing to a greater discrepancy between current standards and the model’s suggestions for female zinc intake.

## Discussion

### Nutrient Intake Analysis

For individuals aged 19 to 70, the RDA for calcium is set between 1000 and 1200 mg/day [34], while the WHO suggests a daily protein consumption of 0.83 g/kg [35], and in Colombia, it is 1.11 g/kg [33]. For example, adults weighing between 50 and 70 kg should aim to consume 42 to 58 g of protein per day, respectively. Our dietary intake analysis (Table 1) reveals that the median calcium intake of the Colombian sample is 284.7 mg/day, significantly below the recommended range, indicating a widespread deficiency in this crucial nutrient. In contrast, the median protein intake is 47.7 g/day, which is within the nutritionally sufficient range.

Notably, the median phytate intake for the total population is reported as 487.0 mg/day (Table 1). This level is significantly lower than the 710 mg/day reference value reported by Larvie and Armah [36] in the USA (*p* < 0.001, Wilcoxon one-sample test). It is pertinent to mention that there is no RDA for phytate, with the average daily intake in the Western diet ranging between 250 and 800 mg [37], aligning with our sample’s intake. These insights are valuable as they provide context for the potential dietary limitations affecting zinc absorption in the Colombian population.

### Analysis of the Phytate-to-Zinc Molar Ratio

An essential aspect in assessing zinc adequacy in the diet is understanding the impact of the P/Z molar ratio on zinc bioavailability. Conducting a Wilcoxon one-sample test revealed that the median P/Z molar ratios in our studied population were significantly below the critical threshold of 15 (*p* < 0.001), suggesting that zinc absorption might not be severely impaired despite the presence of dietary phytate. This observation aligns with previous research indicating significant reductions in zinc absorption for P/Z ratios exceeding 15 [38]. These findings underscore the complex relationship between dietary zinc and phytate, highlighting the need for considering both the quantities consumed and their specific interaction to ensure optimal zinc absorption and inform effective dietary guidelines.

### Understanding Zinc Absorption in the Colombian Population

This study presents a critical evaluation of zinc absorption in the Colombian adult population by employing the 2013 Miller et al. model [31], considering the influences of dietary protein and calcium on zinc bioavailability. Nutritionists face the considerable challenge of accurately determining nutrient absorption from a mixed diet [39] a complexity that our study acknowledges and addresses with this refined model. This model surpasses earlier ones by embracing the intricate dynamics of nutrient interactions within the human digestive system and factoring in the synergistic effects of these nutrients on zinc metabolism [29, 30, 40].

In Colombia, the lack of serum zinc level measurements in the adult population from national surveys, historically limited to children, underscores a significant gap in nutritional data. Our study addresses this void and provides novel insights with the potential to inform public health interventions and nutritional guidelines for adults [16, 17].

The protein intake in adults may offer protection against inadequate zinc absorption [22]. The inclusion of protein, particularly from animal sources, is crucial as it enhances zinc absorption through amino acids that bind zinc, such as histidine and methionine, thereby increasing its solubility and uptake [22]. Additionally, the model’s incorporation of calcium’s competitive inhibition is vital in phytate diets [22, 29], which are characteristic of Colombian diets (see Table 1). By leveraging this comprehensive model, our study not only assesses zinc absorption but also contributes to the advancement in modeling techniques, offering a more accurate reflection of the complexities inherent in zinc metabolism.

Our findings on the FAZ underscore the complexity of zinc absorption in the Colombian adult population. The broad distribution of FAZ values, ranging from 8.9 to 84.8%, not only underscores significant variability in zinc bioavailability among individuals but also reflects the nuanced nature of zinc absorption dynamics observed across different studies. This variability, while initially appearing to exceed the commonly cited range of 16–50% for fractional absorption [8], is in fact aligned with the findings from other studies, such as those by Hunt et al. [41], which have documented a similar wide range of absorption efficiencies within populations. Such observations emphasize the variability in human zinc nutrition and support the need for considering a broader spectrum of individual differences in dietary zinc utilization. The detailed presentation of FAZ metrics, including minimum, maximum, mean, and standard deviation, enriches our understanding of this variability and offers valuable insights for future research and the development of dietary guidelines that account for these variances.

### Reevaluation of Dietary Zinc Recommendations

Utilizing the Miller et al. [31] model, our analysis reveals a significant discrepancy between the zinc intake levels in the Colombian population and the ideal absorption rates according to international standards. This disparity is evident in the TAZ, which falls short of the RAZ as outlined by the IOM (Table 3). A parallel trend is seen in Table 2, where a substantial portion of the population fails to meet either the IOM or Colombian zinc intake standards. Furthermore, Table 4 highlights a disparity between the model’s projections and current guidelines, suggesting that the elevated EAR and RDA estimations provided by the Miller et al. model might more accurately represent the nutritional needs of Colombians. However, it should be noted that while the median RDA_M for men aged 51–70 years coincides with the Colombian RDA, our findings indicate significant variability in the distribution of these values. Therefore, even in this age group where the median aligns with current recommendations, the significant statistical variation observed (*p* < 0.001, Wilcoxon one-sample test) suggests a possible underestimation of the RDA for some individuals, indicating areas where existing guidelines might need adjustment to reflect the actual nutritional needs more accurately.

This inconsistency necessitates a reconsideration of current zinc intake guidelines, potentially warranting an adjustment to align with Miller’s model, particularly for women. Our findings emphasize the importance of adopting varied methodologies and considering regional dietary habits in shaping public health nutrition policies.

Finally, our study sheds light on the complexities of nutrient absorption, especially zinc, in Colombia’s adult population. While employing the Miller et al. [31] model, primarily developed for a US context, revealed significant deficiencies in zinc absorption, it also highlighted the model’s limitations when applied to a Colombian dietary framework. Notably, the model focuses on the impact of phytate, protein, and calcium on zinc absorption but does not encompass other critical factors such as diminished gastric acidity and dietary shifts, particularly relevant for older adults [42]. Additionally, the variability of phytate content from diverse Colombian food sources and preparation methods may have affected the accuracy of our zinc absorption estimates. These findings underscore the need for comprehensive models that consider a broader spectrum of dietary influences, tailoring nutritional interventions to specific population needs and addressing the intricate interplay of various dietary components in zinc absorption.

## Conclusions

This investigation has highlighted the complex interplay between dietary factors and zinc absorption in the Colombian adult population. Utilizing the Miller et al. (2013) model, our study has revealed significant shortcomings in the absorption of zinc, challenging the current RDA and EAR for this vital micronutrient. Furthermore, an analysis of dietary intake patterns separately indicated deficiencies in zinc consumption. These findings underscore the need for age-related dietary adjustments and the consideration of unique Colombian dietary patterns to develop more effective nutritional guidelines.

Future research should focus on longitudinal studies to observe the long-term impact of these adjustments and expand to include bioavailability studies, which could further refine the application of the Miller model. Ultimately, our study advocates for a more tailored approach to dietary recommendations that specifically address the nutritional needs of the Colombian population, aiming to improve zinc nutritional status and overall public health.

## Data Availability

The datasets from ENSIN 2005, filtered and analyzed during the current study, are available upon permission from the Ministry of Health and Social Protection (Ministerio de Salud y Protección Social) in Colombia. For access, please contact via email: repositorio@minsalud.gov.co

## Abbreviations

EAR: Estimated average requirement
ENSIN: Encuesta Nacional de Situación Nutricional (in English, National Nutritional Situation Survey)
FAO: Food and Agriculture Organization
FAZ: Fraction of absorbed zinc
IOM: Institute of Medicine
IZiNCG: International Zinc Nutrition Consultative Group
NHANES: National Health and Nutrition Examination Survey
P/Z: Phytate-to-zinc molar ratio
RAZ: Required absorbed zinc
TAZ: Total absorbed zinc
TDC: Total daily calcium
TDP: Total daily phytate
TDPr: Total daily protein
TDZ: Total daily zinc
RDA: Recommended dietary allowance
WHO: World Health Organization
